# Cardiac Magnetic Resonance for Diagnosis of Neuroendocrine Tumor Metastases to the Right and Left Ventricles with Carcinoid Heart Disease

**DOI:** 10.1155/2019/8746413

**Published:** 2019-12-05

**Authors:** Daniel Barnebee, Brian Morse, Jonathan R. Strosberg, Marijan Pejic, Daniel Jeong

**Affiliations:** ^1^University of South Florida College of Medicine, Tampa, FL, USA; ^2^Department of Diagnostic Imaging and Interventional Radiology, H. Lee Moffitt Cancer Center and Research Institute, Tampa FL, USA; ^3^Department of Gastrointestinal Oncology, H. Lee Moffitt Cancer Center and Research Institute, Tampa FL, USA; ^4^Department of Radiology, University of South Florida College of Medicine, Tampa, FL, USA

## Abstract

A 76-year-old male with a small bowel neuroendocrine tumor with hepatic metastases presented with new onset lower extremity swelling, bloating, and weight gain which ultimately lead to cardiac magnetic resonance (CMR) to evaluate for cardiac involvement of disease. CMR showed right and left ventricular myocardial metastases along with findings suggestive of carcinoid heart disease. The patient had severe tricuspid valve regurgitation necessitating surgical valve repair. The patient underwent bioprosthetic tricuspid valve replacement and debulking of the metastases with surgical pathology confirming neuroendocrine tumor metastases. Follow-up clinical evaluations at 3, 6, and 9 months postoperatively showed improvement in cardiac function and stable hepatic tumor burden. This case demonstrates the utility of CMR to diagnose myocardial metastases and carcinoid heart disease complicated by severe tricuspid regurgitation, which guided surgical management.

## 1. Introduction

Neuroendocrine tumors (NETs) are a diverse group of tumors arising from neuroendocrine cells, and they can cause primary tumors in many different organs including the lungs, small intestine, rectum, pancreas, and other organs [1]. Within this spectrum of disease, neuroendocrine tumors can range from indolent disease to highly aggressive carcinomas. While NETs have an overall incidence of 5.86 per 100,000 people, NETs of the GI tract and lungs (carcinoid tumors) are a type of neuroendocrine tumor with an annual incidence of 4.7 per 100,000 people [2–5]. The most common primary sites of a carcinoid tumor include the small intestine, rectum, and appendix, and hepatic metastases are present in 45% of cases at the time of initial diagnosis [6].

NETs of the midgut (distal small intestine) commonly release vasoactive substances including 5-hydroxytryptamine (serotonin), histamine, tachykinins, and prostaglandins [7]. When these vasoactive substances escape hepatic degradation, the resultant carcinoid syndrome manifests as facial flushing, secretory diarrhea, and bronchoconstriction [2, 7–9]. Secretion of serotonin and other vasoactive substances into the systemic circulation can also result in carcinoid heart disease, a condition characterized by fibrotic damage to the right heart valves (tricuspid and pulmonary). Patients with symptoms of florid carcinoid syndrome have a 50% chance of developing carcinoid heart disease [7].

In rare cases, NETs can metastasize to the heart [10, 11]. Cardiac metastases have been reported as myocardial nodules or pedunculated masses extending into the cardiac chamber, and these can mimic myxomas on echocardiography [12]. NET cardiac metastases often occur after hepatic metastases and have been associated with pericardial effusions [10, 13]. Rare cases of NET metastasizing to the heart have been reported, and cardiac MRI findings of carcinoid cardiac metastases are rarely presented [10, 14, 15]. No specific cases of RV myocardial metastasis extending to the tricuspid valve annular region have been reported.

## 2. Case Presentation

**Figure 1:**
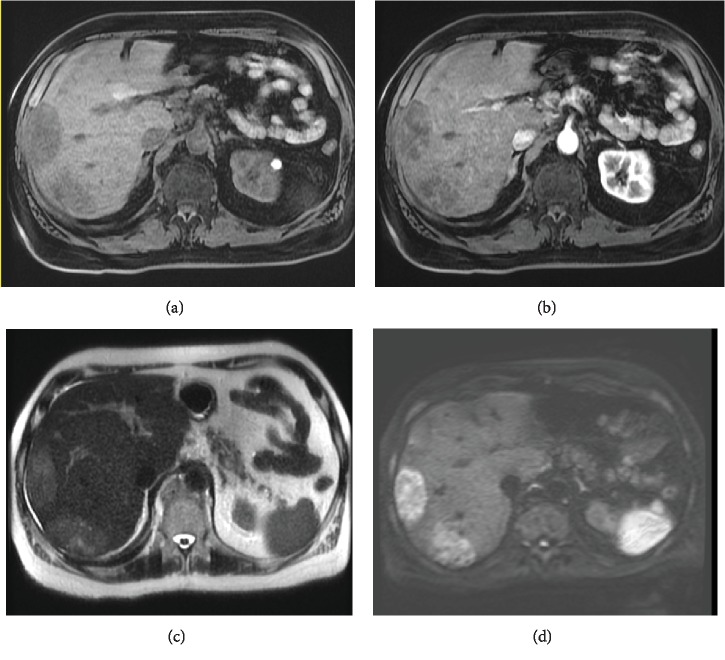
Hepatic metastases on MRI. (a) Axial T1-weighted fat-suppressed precontrast image and (b) postcontrast arterial phase image demonstrating multiple right hepatic lesions with internal enhancement. (c) Axial T2-weighted image shows mildly high signal within the hepatic lesions. (d) Axial diffusion weighted image (*B* = 800) with high signal within the right hepatic lesions compatible with restricted diffusion.

A 76-year-old male with stage IV, small bowel NET metastatic to the liver had been followed by our institution for 4 years with stable hepatic metastatic disease on octreotide long-acting release (LAR) therapy. Originally, the tumor Ki-67 index was 5% and 24-hour urine 5-hydroxyindole acetic acid (5-HIAA) was 105 mg. The patient presented with mild progression of disease on abdominal magnetic resonance imaging (MRI) (Figure 1) after approximately 4 years of stable disease. Six months following this abdominal MRI, the patient developed bilateral lower extremity swelling, weight gain, and bloating lasting over 1 month. The serum brain-natriuretic peptide (BNP) value was 161 pg/ml (normal range < 100 pg/ml). N-terminal proBNP and other cardiac biomarkers were not available. Transthoracic echocardiogram showed tricuspid regurgitation and right ventricular wall thickening with an echogenic mass in the RV free wall extending to the RV outflow tract. There was severe malcoaptation of the thickened tricuspid valve leaflets. Tricuspid regurgitation was described as widely open regurgitation with a peak regurgitant flow velocity of 203 cm/s. Of note, the aortic and mitral valves were structurally normal without stenosis or regurgitation. Cardiac magnetic resonance (CMR) was obtained for further evaluation.

**Figure 2:**
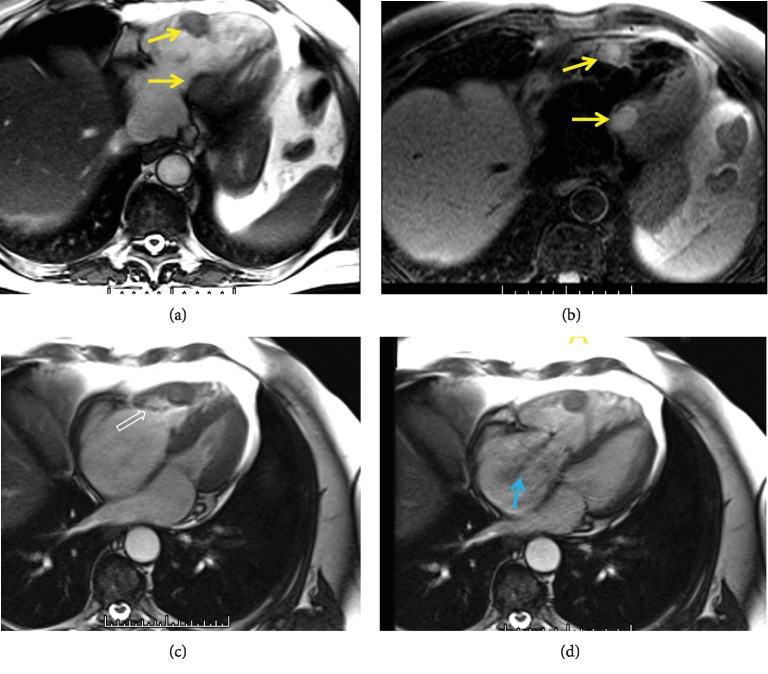
(a) Axial bSSFP bright blood image through the right ventricle (RV) shows two masses (yellow arrows) involving the anterior RV wall and basilar interventricular septum. (b) Axial T2 spectral presaturation with inversion recovery dark blood image showing T2 hyperintense signal within the two lesions (yellow arrows). (c) Four-chamber bSSFP bright blood image during late systole shows thickening of the anterior leaflet of the tricuspid valve (open arrow) with an abnormal shortened and retracted configuration near the anterior RV wall mass. (d) Early systole image shows a large low signal tricuspid regurgitant flow jet (blue arrow). Notice the anterior leaflet maintaining an abnormal partially open configuration.

CMR (Figure 2) showed a 2.6 cm lesion in the right ventricular free wall and a 2.4 cm lesion in the left ventricular anteroseptal wall. These lesions demonstrated high T2-weighted signal and isointense T1-weighted signal with postcontrast perfusion, findings compatible with myocardial metastases. Of note, the RV free wall lesion extended superiorly near the base of the tricuspid valve annular region. Balanced cine steady state free precession (bSSFP) images showed abnormal thickening of the anterior tricuspid valve leaflet, and the leaflet did not coapt normally during systole contributing to severe tricuspid regurgitation. The right atrium was moderately dilated.

The patient underwent bioprosthetic tricuspid valve replacement and biopsy and debulking of the myocardial tumors. The myocardial tumors were histologically confirmed as NET metastases. The patient recovered well from surgery, and follow-up clinical visits demonstrated significant improvement in his carcinoid heart disease. As of the latest visit, the patient has excellent cardiac function with resolution of tricuspid regurgitation. His 9-month postoperative follow-up CT scan showed relatively stable disease, and his urine 5-HIAA was improving. The patient is continuing on octreotide LAR 30 mg.

## 3. Discussion

We present a unique case of carcinoid heart disease and myocardial metastases both contributing to this patient's severe symptomatic tricuspid regurgitation. The tricuspid valve thickening is a specific finding of carcinoid heart disease, and well-defined lesions with arterial perfusion in the RV and LV walls are specific for metastases. Previous authors have suggested that vasoactive mediators associated with myocardial metastases may accelerate the onset of carcinoid heart disease, which could explain the case described here [12, 16].

Carcinoid heart disease manifests via the accumulation of carcinoid plaque composed of smooth muscle, myofibroblasts, and elastic tissue [3]. Plaque forms a fibrous layer lining the endocardial surface of the valves thus displacing the fibrous tissue on the endocardial surfaces of the heart. Morphologic changes to the heart including dilatation of valve rings can lead to tricuspid or pulmonic valve insufficiency and ultimately right heart failure, while the left heart is involved in less than 10% of cases [3, 13]. When the left heart is involved in carcinoid heart disease, extensive hepatic metastases, bronchial carcinoid, or a patent foramen ovale may be present [17, 18]. Myocardial NET metastases, however, often present as discrete lesions within the right or left ventricular myocardium. In our case, the discrete RV metastasis adjacent to the tricuspid valve annular region likely accelerated carcinoid heart disease due to local tumor release of vasoactive substances including serotonin.

Echocardiography serves as first line screening for cardiac involvement in patients with carcinoid syndrome. Common echocardiographic features of carcinoid heart disease include morphologic changes to the tricuspid valve causing a thick retracted appearance with decreased mobility and enlargement of the right atrium and ventricle related to valvular insufficiency [19]. Surgical valve replacement is considered when patients are symptomatic with severe valvular dysfunction or when RV function significantly declines due to valvular disease [8].

CMR offers more specific evaluation of cardiac lesions to help differentiate benign lesions from metastases compared to echocardiography evaluation which is based on lesion intrinsic signal and perfusion characteristics [20]. While echocardiography allows for excellent real-time myocardial visualization with flow information, the CMR T2-weighted and postcontrast sequences allow for more specific mass characterization [13]. Cardiac metastases can affect the right or the left side of the heart, as well as the ventricular septum, and appear continuous with the affected myocardial wall [3, 11, 16]. CMR images in this case show the right ventricular myocardial tumor in close proximity to the tricuspid valve with markedly abnormal tricuspid valve closing. The anterior tricuspid valve leaflet had a shortened retracted configuration. It is likely that the close proximity of the RV myocardial metastasis accelerated valve leaflet morphological changes associated with carcinoid heart disease.

This case demonstrates the importance of CMR to evaluate for carcinoid heart disease and cardiac metastases. The results of the CMR exam in our case lead to characterization of myocardial metastases and allowed for planning of metastasis resection during the tricuspid valve bioprosthetic surgery. The case also serves as a reminder for providers that while echocardiography may offer first line screening for cardiac involvement when carcinoid heart disease is suspected, CMR may be useful when myocardial metastases are suspected for potential surgical planning.

## Supplementary Materials

Supplementary Materials Figure 3 (supplemental video clip): cine bSSFP 4 chamber imaging demonstrates severe tricuspid regurgitation and abnormal failure of the tricuspid valve to coapt or close during systole. The anterior right ventricular wall metastasis is also noted.
